# Differential scaling within an insect compound eye

**DOI:** 10.1098/rsbl.2016.0042

**Published:** 2016-03

**Authors:** Craig D. Perl, Jeremy E. Niven

**Affiliations:** School of Life Sciences, University of Sussex, Falmer, Brighton BN1 9QG, UK

**Keywords:** scaling, wood ant, *Formica rufa*, grade shift, slope shift, facet diameter

## Abstract

Environmental and genetic influences cause individuals of a species to differ in size. As they do so, organ size and shape are scaled to available resources whilst maintaining function. The scaling of entire organs has been investigated extensively but scaling within organs remains poorly understood. By making use of the structure of the insect compound eye, we show that different regions of an organ can respond differentially to changes in body size. Wood ant (*Formica rufa*) compound eyes contain facets of different diameters in different regions. When the animal body size changes, lens diameters from different regions can increase or decrease in size either at the same rate (a ‘grade’ shift) or at different rates (a ‘slope’ shift). These options are not mutually exclusive, and we demonstrate that both types of scaling apply to different regions of the same eye. This demonstrates that different regions within a single organ can use different rules to govern their scaling, responding differently to their developmental environment. Thus, the control of scaling is more nuanced than previously appreciated, diverse responses occurring even among homologous cells within a single organ. Such fine control provides a rich substrate for the diversification of organ morphology.

## 1. Introduction

In natural environments, adults from a single species can vary enormously in body size owing to a combination of genetic and environmental factors. Organ size changes to accompany changes in body size, a process known as allometric scaling [1,2]. Theories of organ scaling [2,3] have focused on entire organs and how their relative proportions change with whole body size, largely ignoring changes in the size and number of cells within organs (but see [4]). Here, we investigate scaling within an organ, the compound eye of an insect.

Organ scaling has been studied in numerous taxa but particularly in holometabolous insects [5–9] because the organs of adults of these insects develop during pupation from cellular monolayers, called imaginal discs [10,11]. Insect compound eyes provide an opportunity to explore scaling within an organ because the facet array provides a read-out at cellular-level resolution of relative investment in individual facets [4,12]. During development individual retinal cells arise from an ommatidial progenitor [13,14] and do not contribute to adjacent ommatidia as they differentiate [15]. Therefore, facet scaling provides some information about resource allocation at the cellular level within an imaginal disc during development.

We studied the scaling of wood ant (*Formica rufa* L.) worker compound eyes. The area of their compound eyes as well as the numbers of facets and their diameters increase with body size, though they do so with negative allometry. We found substantial heterogeneity in scaling of facet diameter between different regions of the compound eye, demonstrating hitherto unknown control of scaling of structures within organs.

## 2. Material and methods

### (a) Animals

*Formica rufa* (Hymenoptera: Formicidae) colonies were collected from Ashdown Forest, Sussex, UK (N 51 4.680, E 0 1.800) between June 2013 and August 2014, and maintained indoors at 21°C under a 12 L : 12 D cycle.

### (b) Specimen preparation

Individual ants were restrained and transparent nail varnish (Rimmel London, UK) was applied to both compound eyes to create a cast. Once dried, the casts were removed, flattened and mounted onto 12.5 mm specimen stubs (Agar Scientific, UK). The rear left femur of each ant was used as a proxy for the size of the ant [16]. Specimens were imaged using a scanning electron microscope (S420 Stereoscan, LEO Electron Microscopy Ltd., Germany).

### (c) Measurements

Nine facet diameters from four separate eye regions were selected at random and measured from 66 ants (2376 facets in total) from three colonies. The diameters of every facet from a representative small and large ant were measured to produce eye ‘heatmaps’. Diameters were measured from scanning electron micrographs using ImageJ v. 1.48 [17].

### (d) Statistics

Statistics were calculated using R v. 3.1.2. [18]. Facet diameter scaling was investigated with linear mixed effect models by using the lme function from the ‘nlme’ package [19]. Custom contrast matrices were used to make *post hoc* multiple pair-wise comparisons (*t*-tests) of linear mixed effect models with the estimable function from the ‘gmodels' package [20].

## 3. Results

**Figure 1.**
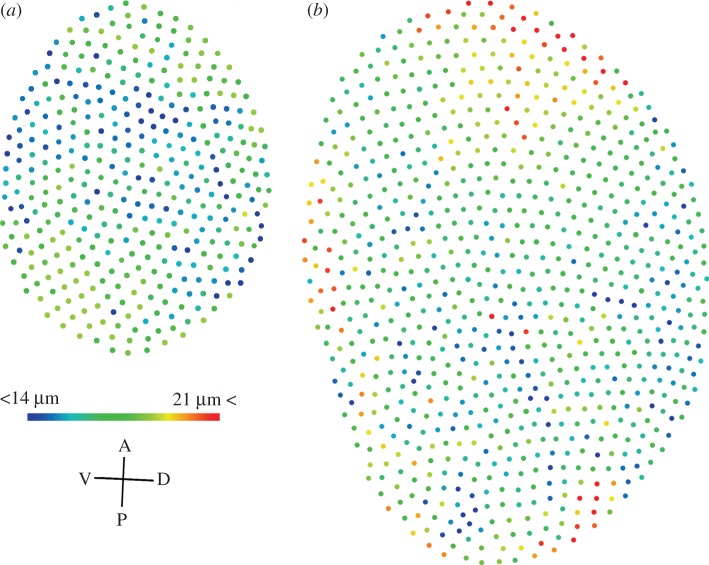
Regional differences exist in the diameters of facets from compound eyes of wood ant workers. (*a*) A heat-map of the diameter of each facet from a single wood ant worker compound eye, 382 facets in total. (*b*) As in (*a*) but for an eye from a larger worker, 815 facets in total. A, P, V, D: anterior, posterior, ventral, dorsal.

We measured the facet diameters of the eyes of small and large workers (figure 1), creating maps of facet diameters [21]. These maps revealed differences in facet diameters between the large and the small workers, as well as regions of the eye in which facets differed systematically in diameter. In the eye of the larger worker, the largest facets are found mainly in the anterior–dorsal region, whereas in the smaller worker eye, the largest facets are restricted to the anterior–dorsal and ventral–posterior regions (figure 1). To quantify differences in facet diameter between regions and across a range of worker body sizes, we measured facet diameters from four regions (anterior, posterior, ventral and dorsal) of the compound eye.

**Figure 2.**
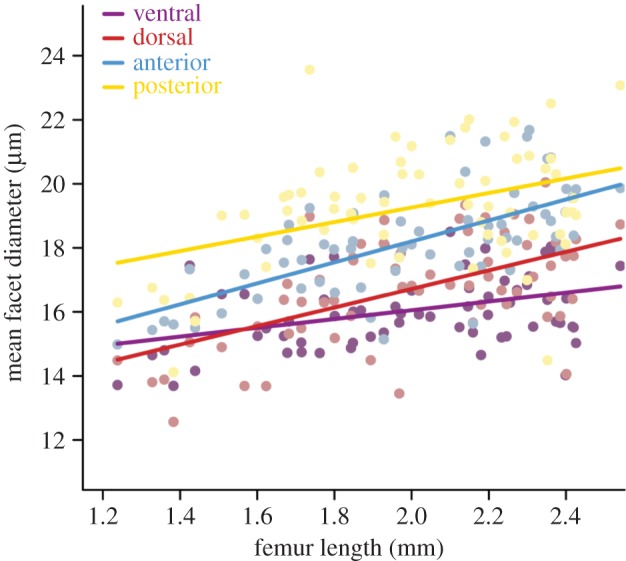
The diameters of facets from different eye regions scale differentially. The graphs show the scaling of facets from the anterior, posterior, dorsal or ventral regions of the compound eye for workers from three wood ant nests.

Comparisons among eye regions showed that, for a given body size, facets differed in absolute diameter between regions within an individual ant. Facet diameters were, however, larger across all regions of the larger worker eyes than in those of smaller workers. The intercept of the posterior region was significantly higher than that of the anterior region (*t*_65,188_ = 2.69, *p* = 0.008). The dorsal region had a significantly lower intercept than either the posterior (*t*_65,188_ = 3.28, *p* = 0.001) or ventral eye regions (*t*_65,188_ = 2.05, *p* = 0.04). There were no differences in the intercepts of the remaining regions (*t*_65,188_ < 1.47, *p* > 0.1). Thus, as workers increase in size, the facets of some regions increase in diameter at the same rate, producing grade shifts in their allometric scaling relationships (figure 2).

Comparison among eye regions also revealed significant differences in the slope of the scaling relationship, the rate at which facet diameter increased with increasing body size. The facet diameters in the anterior (*t*_65,188_ = 3.36, *p* = 0.001) and dorsal regions of the eye (*t*_65,188_ = 2.65, *p* = 0.009) increased with body size at a faster rate than those from the ventral eye region. There were no slope differences between the remaining regions (*t*_65,188_ < 1.81, *p* > 0.07). Thus, as workers increase in size, facets in the anterior–dorsal region increase in diameter at a greater rate than the rest of the eye.

## 4. Discussion

Comparison of facet diameters among different regions of the wood ant compound eye shows that they scale heterogeneously. In some regions, large facets increase in diameter at the same rate as other absolutely smaller facets in other regions, indicative of grade shifts. This implies that facets in these regions are of equal importance, and that additional resources associated with increased body size are allocated proportionately. Some regions differ in slope of their scaling relationships, showing that available resources are disproportionately allocated within the developing eye imaginal disc, larger individuals investing more in the anterior and dorsal regions of the compound eye than the ventral region.

### (a) Proximate mechanisms

We propose that individual cells within an imaginal disc use nutrients to different extents. Growth and nutrition are linked by insulin production [22,23]. Cells may show regional differences in their expression of insulin receptors, so that when exposed to the same increased levels of insulin-like peptides those that express more insulin receptors will grow at a faster rate. This mechanism is analogous to that proposed to account for the differential growth of imaginal discs underlying exaggerated traits and could provide the basis for the evolution of organ shape changes [3]. One putative mechanism is that adjusting the number of insulin and ecdysone receptors in different parts of an imaginal disc could alter the shape of an entire organ. Such changes could, for example, contribute to the evolution of the horns of adult males from different species of *Onthophagus*, which differ in the number of prongs and their shape [24].

### (b) Functional implications

Increases in facet diameter improve sensitivity by improving photon capture [25]. Thus, differences in facet diameter within the wood ant compound eye are presumably a consequence of needing regions of high sensitivity and resolution with limited resources and space available. Such specialized regions are common in compound eyes and are typically associated with specific aspects of behaviour where high performance is required, such as mate or prey detection [25]. However, previous studies have not considered that particular regions of the compound eye may differ from one another in terms of their scaling [26].

Slope shifts indicate differential investment in particular regions depending on body size. Such differential investment may be related to task differentiation; despite lacking distinct morphological castes, larger ants forage further from the nest than smaller ants [27], which may necessitate greater investment in vision. However, rhabdom structure and inter-ommatidial angles are needed to determine the impact of these differences in scaling upon wood ant vision.

More generally, increased investment in specific regions of the compound eye or other sensory structures may confer an advantage on larger individuals of a particular species in specific tasks, especially when such regions are linked to the detection of mates or prey. This raises the possibility that eye regions such as the love spot of male houseflies [28] may also show scaling indicative of greater investment in larger individuals. This would produce exaggerated sensory structures analogous to the exaggerated morphological traits more typically associated with sexual selection, such as *Onthophagus* beetle horns [24]. However, the lattice structure of the compound eye may constrain investment in such regions, preventing them from showing the extreme positive allometry of beetle horns.

## Data accessibility

Data deposited in Dryad: http://dx.doi.org/10.5061/dryad.t5h1j.
